# Thrombospondin-1 Is a Putative Target Gene of Runx2 and Runx3

**DOI:** 10.3390/ijms140714321

**Published:** 2013-07-10

**Authors:** Xiuming Shi, Vishwa Deepak, Linghui Wang, Xueqing Ba, Toshihisa Komori, Xianlu Zeng, Wenguang Liu

**Affiliations:** 1 Institute of Genetics and Cell Biology, Northeast Normal University, Changchun 130024, China; E-Mails: xiumingbio@126.com (X.S.); viishwadeepak@gmail.com (V.D.); linghuibio@126.com (L.W.); baxq755@nenu.edu.cn (X.B.); zengx779@nenu.edu.cn (X.Z.); 2 Division of Surgery, Indian Veterinary Research Institute, Izatnagar 243122, India; 3 School of Dentistry, Nagasaki University, 1-7-1 Sakamoto, Nagasaki 852-8588, Japan; E-Mail: komorit@nagasaki-u.ac.jp; 4 Key Laboratory of Molecular Epigenetics of Ministry of Education of China, Northeast Normal University, Changchun 130024, China

**Keywords:** TSP-1, Runx2, Runx3, C3H10T1/2 stem cells, osteoblast, metastasis, cancer, melanoma, B16-F10, angiogenesis

## Abstract

Thrombospondin-1 (TSP-1), a matricellular protein widely acclaimed to be involved in the inhibition of angiogenesis and tumorigenesis, is synthesized and secreted by many cell types, including osteoblast and cancer cells. TSP-1 is highly upregulated during early stage of osteogenesis, whereas it inhibits terminal osteoblast differentiation. Expression of TSP-1 is downregulated in cancer cells, and its ectopic expression has been shown to restrain tumor growth. Transcriptional regulation of TSP-1 in osteogenesis and cancer is poorly understood; this prompted us to study its regulation by the two key regulators of the aforementioned processes: Runx2 and Runx3. Through a PCR-based cDNA subtraction technique, we identified and cloned a cDNA fragment for mouse TSP-1, whose expression was dramatically upregulated in response to Runx2 expression in mesenchymal stem cells. Moreover, TSP-1 expression was considerably reduced in the lung of Runx2 knockout mouse. On the other hand, TSP-1 gene expression drastically increased at both the transcriptional and translational levels in response to Runx3 expression in B16-F10 melanoma cells. In line with this, Runx2 and Runx3 bound to the TSP-1 promoter and stimulated its activity. Hence, these results provide first line of evidence that TSP-1 is a transcriptional target gene of Runx2 and Runx3.

## 1. Introduction

Thrombospondins are extracellular calcium binding glycoproteins that belong to a small family of proteins comprised of five members: TSP-1 to TSP-5. TSPs are encoded by separate genes, where TSP-1 and TSP-2 (their protein products appear as homotrimers) belong to group-A; on the other hand, TSP-3 to TSP-5 (their protein products appear as homopentamers) belong to group-B [1]. TSP-1 is a 450 kDa multimeric glycoprotein originally studied as a protein released from thrombin stimulated platelets; however, it is now reported that TSP-1 is expressed by many cell types, such as mesenchymal cells, tumor cells, fibroblasts, osteoblasts, adipocytes, endothelial cells, smooth muscle cells, monocytes and macrophages [2–6]. TSP-1 can interact simultaneously with various integrins and non-integrins, matrix proteins and cytokines, allowing crosstalk between cell surface receptors to regulate a plethora of processes, like adhesion, migration, metastasis, angiogenesis, cell proliferation and differentiation [7–9].

TSP-1 is a potent angiogenesis regulator and is the first endogenous antiangiogenic factor to be reported [10]. Expression of TSP-1 in various cancers is significantly downregulated in comparison to normal cells, whereas, re-expression of TSP-1 in tumor cells has been shown to restrain cancer growth by 50% to 75% [11]. TSP-1 overexpression in mice has been shown to suppress wound healing and tumorigenesis, while lack of TSP-1 increased vascularization of tissues [12,13]. Additionally, expression of TSP-1 is reported to be negatively modulated by oncogenes and maintained by tumor suppressors [14]. TSP-1 has been shown to recruit tumor activated macrophages to target cancer cells and has been suggested to be a potential therapeutic target against cancer [15,16]. Thus, expression of TSP-1 might play different regulatory roles in normal and transformed cell lines, and understanding of TSP-1 transcriptional regulation in these cells would be very much beneficial in designing therapies that target TSP-1 expression.

TSP-1 is expressed in the odontoblasts, developing skeleton, long and calvarial bones [2,17–19]. TSP-1 is a major constituent of bone matrix proteins that is synthesized and secreted by osteoblasts [18]. TSP-1 is highly upregulated during osteogenic differentiation of MC3T3-E1 preosteoblasts with an increase of 10- to 15-fold and coincides with the expression of early osteogenic marker, alkaline phosphatase (ALP), which, later on, decreases at basal levels during terminal differentiation of osteoblasts [2]. TSP-1-null mice show craniofacial dysmorphism [20]. On the other hand, TSP-1 has been reported to inhibit bone nodule formation, osteoblast differentiation and endochondral ossification [21,22]. Recent findings suggest that TSP-1 can inhibit late stage osteoblast differentiation by activation of latent TGF-β in human mesenchymal stem cells [23]. Dissimilar patterns of expression and contrasting roles of TSP-1 indicates dual pattern of regulation during osteogenesis and warrant further investigation of its transcriptional regulation in osteoblasts.

The Runt-related transcription factors (Runxs) belong to a family of evolutionarily conserved proteins, which share the highly homologous DNA-binding, N-terminal Runt domain of about 120 amino acids [24]. To date, three Runx transcription factors, *i.e.*, Runx1, Runx2 and Runx3, have been identified, which regulate diverse biological processes and show differential, tissue-specific expression patterns. Runx1 regulates hematopoiesis, Runx2 is the master switch of osteoblast differentiation and controls bone formation and Runx3 is necessary for gastric epithelial growth, lung epithelial cell differentiation and neurogenesis [25]. Runx3 is a major tumor suppressor gene and is frequently lost in various types of cancer (lung, gastric, colorectal, breast, bladder and melanoma), due to hemizygous deletions or epigenetic alterations. Accumulating reports suggest that Runx3 is critical for various melanomas [26,27]. Runx2 is a key regulator of osteoblast differentiation and function. Mutations in Runx2 lead to Cleidocranial dysplasia syndrome, and lack of Runx2 results in complete absence of bone formation, whereas overexpression of Runx2 results in osteopenia and blunted terminal differentiation of osteoblasts and mineralization [28–30]. Runx2 regulates the expression of several bone matrix protein genes in osteoblasts, such as type I collagen, osteopontin, osteocalcin, collagenase 3 and SPARC (Secreted Protein, Acidic, Cysteine-Rich) [31].

TSP-1 is a constituent of bone matrix proteins that is synthesized and secreted by osteoblasts. TSP-1 is highly expressed at the onset of osteogenic differentiation; however, its expression falls down at the terminal stage of osteoblastogenesis. Constitutive expression of TSP-1 leads to inhibition of late stage osteogenesis. Osteogenic differentiation is controlled by Runx2; however, the role of Runx2 in transcriptional regulation of TSP-1 during osteogenesis is unknown. Similarly, TSP-1 is a potent inhibitor of angiogenesis and tumorigenesis; still, the role of Runx3 in transcriptional regulation of TSP-1 in cancer cells is not studied in detail. Here, we studied the two critical Runx transcription factors, Runx2 and Runx3, in regulating the transcription of TSP-1 in osteoblasts and melanoma cells, respectively.

## 2. Results and Discussion

### 2.1. Runx2 Mediated Regulation of TSP-1 Gene Transcription in Mesenchymal Stem Cells

**Figure 1:**
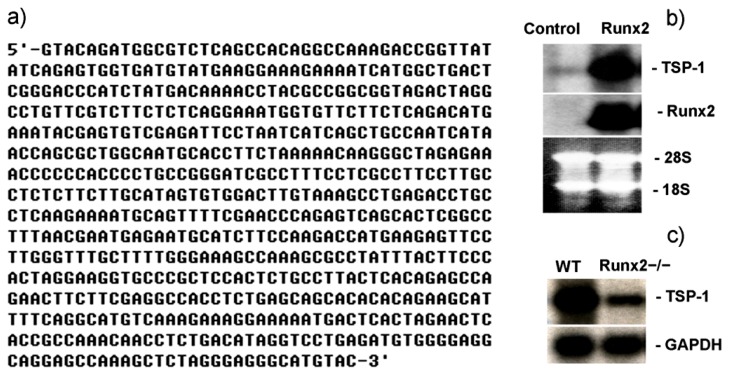
Runx2 regulates TSP-1 expression. (**a**) Sequence of TSP-1 cDNA fragment identified through a cDNA subtraction technique that was upregulated by Runx2; **(b**) Northern blot analysis of TSP-1 expression in Runx2 overexpressing C3H10T1/2 stem cells, where the TSP-1 cDNA fragment was identified via a cDNA subtraction technique and used as a probe, 28S and 18S RNA bands on the membrane were used as control for equal loading; and (**c**) Northern blot analysis of TSP-1 expression in lung tissue of Runx2 knockout mouse, where glyceraldehyde 3-phosphate dehydrogenase (GAPDH) served as internal control.

TSP-1 is an extracellular matrix protein of bone and is expressed at remarkable levels during the early stage of osteoblast differentiation, similar with another marker of osteogenesis, ALP [2]. Due to this feature of TSP-1 it has also been suggested to be considered as an early marker of osteogenic differentiation. Initiation of osteogenesis and expression of osteoblastic genes are regulated and triggered by the master gene of bone formation, Runx2, whose expression alone is adequate enough to launch the expression of genes involved in bone formation [32]. Since TSP-1 expression coincides with the initiation of osteogenic gene transcription, we asked if Runx2 can regulate TSP-1 transcription in osteoblasts. Mouse mesenchymal stem cells C3H10T1/2 overexpressing Runx2 and WT-C3H10T1/2 cells (control) were compared using a cDNA subtraction approach to obtain clones of genes that are differentially expressed in these two populations. One-hundred twenty clones were sequenced and identified through National Center for Biotechnology Information (NCBI) BlastN service. Those appeared more than three times were regarded as candidate target genes of Runx2 for further analysis. Among them, five cDNA sequences solely homologous to the same 3′-UTR (untranslated region) of TSP-1 gene were identified. By using the same cDNA fragment (Figure 1a) as the probe in Northern blotting, we confirmed that the expression of TSP-1 in C3H10T1/2 mesenchymal stem cells was dramatically upregulated by Runx2 (Figure 1b).

To address whether Runx2 is also involved in regulating the gene expression of TSP-1 *in vivo*, we first compared TSP-1 gene expression in the skeletons of Runx2^−/−^ and wild-type (WT) newborn mice by Northern blotting. Unfortunately, we could not find any change in TSP-1 gene expression in Runx2 knockout skeletons as compared to WT (data not shown). This might be due to the fact that Runx2 gene targeting results in no bone formation; however, the mutant skeleton composed only of chondrocytes still expresses TSP-1 and, therefore, may compensate the loss of TSP-1 gene expression accompanied with the loss of osteoblasts in Runx2-deficient skeleton [28]. Since lung is another tissue that expresses Runx2 [33] and the organogenesis of lung is normal in Runx2 knockout mice, we observed that the expression of TSP-1 in lung was considerably reduced by the deletion of Runx2, as compared to the TSP-1 expression in WT-mouse (Figure 1c). These findings strongly indicate that Runx2 regulates TSP-1 gene expression both *in vitro* and *in vivo* and provide a first line of evidence regarding transcriptional regulation of TSP-1 by Runx2.

We have reported elsewhere that overexpression of Runx2 inhibits osteoblast maturation [30], suggesting that the expression levels of Runx2 must be reduced during osteoblast maturation. As TSP-1 can indirectly decrease Runx2 expression [23], and its expression can be upregulated by Runx2 during the initial stage of osteoblast differentiation, whereas late stage expression of TSP-1 can hamper osteoblast mineralization [21]. Similar with the effects mediated by Runx2 [30], it seems that TSP-1 might be exerting a negative feedback impact on Runx2 expression during late stage osteoblast differentiation. Another explanation might be that constantly high expression levels of Runx2 during late stage osteogenesis might maintain TSP-1 expression, due to which low mineralization occurs in Runx2 transgenic mice [30], and also in MC3T3-E1 preosteoblasts constitutively expressing TSP-1 [21]. Further work specifically targeting the Runx2-induced TSP-1 expression at the terminal stage of osteogenic differentiation can shed light on the mechanism of action of Runx2 and TSP-1 at late stage osteoblastogenesis.

### 2.2. Runx3 Mediated Regulation of TSP-1 Gene Expression in B16-F10 Melanoma Cells

Tumors require continuous formation of new blood vessels to promote invasion and nourishment of tumor cells [34]. TSP-1 is a natural inhibitor of angiogenesis [15], and its expression is reported to be frequently downregulated in a wide array of tumors with upregulation of pro-angiogenic factors [14,35]. Expression of TSP-1 suppresses tumor growth *in vitro* and *in vivo* [12,36]. Low levels of TSP-1 expression have been associated with increased recurrence rates and decreased overall survival rates in several human cancers [11], suggesting that the loss of TSP-1 is critical for tumor development. Downregulation of TSP-1 along with accelerated angiogenesis has been a paradigm in various cancers [37]. Oncogenes, such as ras, myc and HER2, tend to downregulate the expression of TSP-1, whereas tumor suppressors, p53, PTEN and Smad4, have been reported to increase TSP-1 expression [15]. Runx3 is a potent tumor suppressor gene, whose downregulation or inactivation results in increased angiogenesis and metastasis in various cancers [38,39], whereas its expression can induce antiangiogenic and antimetastatic phenotype by inhibiting vascular endothelial growth factor (VEGF) [40]. Recently characterized functions of Runx3 include interaction with DNA repair proteins, inhibition of angiogenesis and involvement in cell adhesion and invasion [41]. Analyzing these lines of evidence, it seems plausible that there might be a crosstalk between Runx3 and TSP-1, where Runx3 might modulate the transcriptional regulation of TSP-1 expression. Although, it is clear that Runx3 promotes the inhibition of angiogenesis and tumor growth, respectively, none of the previous studies reported the regulatory effects of Runx3 on TSP-1 expression, a factor critical for angiogenesis and tumorigenesis. Since, Runx3 acts as a tumor suppressor and regulates the processes of angiogenesis and tumorigenesis, we were interested to know if TSP-1 is a downstream target of Runx3.

**Figure 2:**
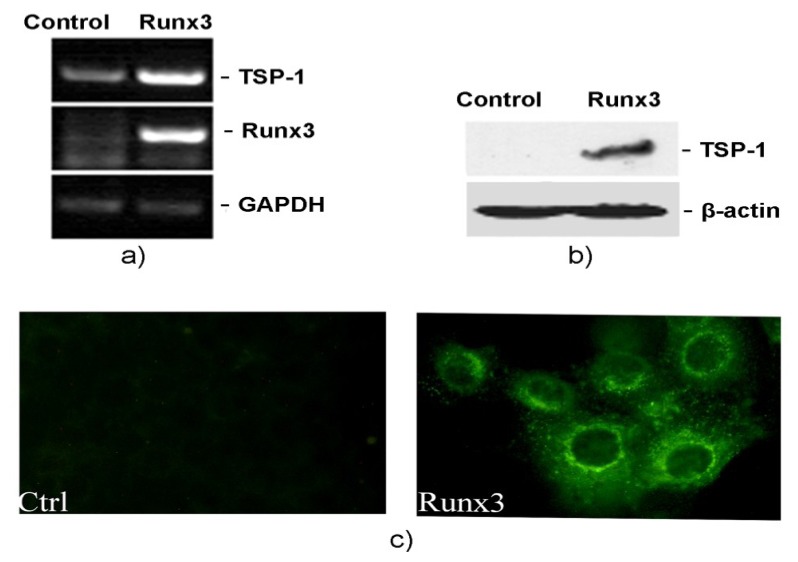
Runx3 regulates TSP-1 expression. (**a**) RT-PCR analysis of TSP-1 expression in B16-F10 melanoma cells infected with empty retrovirus (control) or Runx3 bearing retrovirus with GAPDH as an internal control; (**b**) Western blot analysis of TSP-1 expression in Runx3 overexpressing B16-F10 melanoma cells with β-actin as an internal control; and (**c**) Immunofluorescence analysis of TSP-1 expression in cells lacking Runx3 (Ctrl) and cells expressing Runx3 (Runx3).

To find out if Runx3 can modulate TSP-1 expression, we conducted RT-PCR and Western blot experiments by restoring Runx3 expression in B16-F10 melanoma cells. Ectopic expression of Runx3 resulted in a dramatic increase of mRNA and protein expression levels of TSP-1 (Figure 2a,b). Furthermore, immunofluorescence studies conducted on B16-F10 cells with restored Runx3 expression showed a prominent staining of the induced TSP-1 in cytoplasm, as compared to control cells lacking Runx3 expression (Figure 2c). These results clearly demonstrate that TSP-1 is a putative downstream target gene of Runx3, and upregulating TSP-1 expression levels in cancer cells might be a novel mechanism through which Runx3 exerts its tumor suppressor functions.

TSP-1 indirectly regulates tumor growth by activating TGF-β and inhibiting extracellular MMPs (matrix metalloproteinases) [13,42]. Therefore, TSP-1 can inhibit angiogenesis and tumorigenesis by inducing apoptotic pathways, as well as activation of TGF-β and inhibition of VEGF-activated survival pathways [7]. Runx3 mediated inhibition of cancer involves various pathways [41]; however, Runx3 has been reported to hamper angiogenesis and tumor growth by inhibiting the VEGF pathway in a manner similar with that of TSP-1 [40]. Likewise, accumulating evidence suggests that restoration of Runx3 expression can inhibit angiogenesis and cancer cell migration and invasion [38]; additionally, a study by Lee *et al.* suggests that loss of function of Runx3 in liver led to a marked increase in the expression of VEGF, von Willebrand factor (vWF) and cluster of differentiation 31 (CD31) [39], well-known factors involved in angiogenesis and regulated by TSP-1 [6,9]. These reports illustrate that the mode of action of Runx3 and TSP-1 might be similar in terms of regulating metastasis and angiogenesis. Our finding that Runx3 regulates TSP-1 expression provides a novel outlook and mechanism that lays a foundation for future studies in understanding the biology of TSP-1. Further work, specifically focusing on the Runx3 effect on TSP-1 expression, would certainly be beneficial in designing cancer therapies and further our knowledge on the complex events regulating these diseases.

### 2.3. TSP-1 Gene Promoter Is Regulated by Runx2 and Runx3

**Figure 3:**
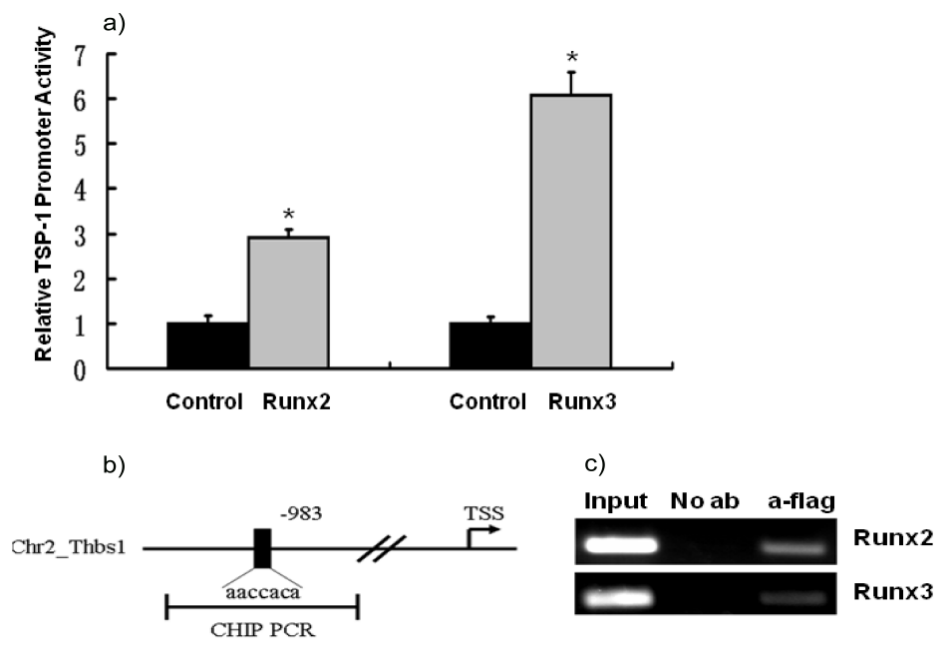
TSP-1 promoter activity is regulated by Runx2 and Runx3. (**a**) NIH3T3 cells were transfected with TSP-1 promoter-luciferase reporter plasmids and Runx2 or Runx3 expression plasmids to analyze TSP-1 promoter activity by luciferase assay. Data were analyzed using the Student’s *t*-test; asterisk denotes significance at *p* < 0.05; (**b**) Schematic diagram illustrating the fragment of the TSP-1 gene that was amplified. The position of PCR primers used to detect the TSP-1 promoter fragment is indicated by the line denoting CHIP PCR; (**c**) Runx2 overexpressing C3H10T1/2 mesenchymal stem cells and Runx3 overexpressing B16-F10 melanoma cells were immunoprecipitated with no antibody or anti-flag antibodies for flag-Runx2 and flag-Runx3, followed by PCR with the primers mentioned in the Materials and Methods.

Since Runx2 efficiently upregulated the expression of TSP-1 evident through cDNA subtraction and *in vivo* assays and Runx3 increased the expression of TSP-1 as seen in RT-PCR and Western Blot experiments, we further asked if Runx2 and Runx3 can stimulate TSP-1 promoter through dual-luciferase promoter assay. NIH3T3 cells were transfected with TSP-1 promoter-reporter plasmids and control or effector plasmids for analyzing TSP-1 promoter activity under the effect of Runx2 and Runx3. TSP-1 promoter activity was significantly stimulated by Runx2 and Runx3 (*p* < 0.05, Figure 3a). To further strengthen our finding that the Runx factors can modulate TSP-1 promoter, we performed CHIP assays. As expected, Runx2 and Runx3 bound to the TSP-1 gene promoter, indicating that TSP-1 is a transcriptional target gene of Runx2 and Runx3 (Figure 3c). These results provide a further line of evidence regarding the regulation of TSP-1 gene expression by Runx3, the tumor suppressor gene, and Runx2, the bone specific transcription factor.

## 3. Experimental Section

### 3.1. Cell Culture

C3H10T1/2 cells stably expressing flag-tagged Runx2, and reported elsewhere [32], were maintained in DMEM with 10% FBS and 500 μg mL^−1^ G418. The Runx2 knockout newborn mice used in the study were described earlier [28]. B16-F10 melanoma cells stably overexpressing flag-tagged Runx3 or their control cells were established as per the previously published protocol [32] and were maintained in Iscove’s Modified Dulbecco’s Medium (IMDM) with 10% FBS and 300 μg mL^−1^ G418. All of the cell lines were incubated at 37 °C in a humidified atmosphere with 5% CO_2_.

### 3.2. cDNA Subtraction

Subtraction of the cDNAs that were upregulated by Runx2 was performed using a PCR-select^™^ cDNA subtraction kit (Clontech, Palo Alto, CA, USA), using the protocol provided with the kit. The starting amount of poly-A RNA was two micrograms each. Tester cDNAs containing specific (differentially expressed) transcripts and driver cDNAs were prepared by digesting double stranded cDNAs with RsaI. Subsequently, only the tester cDNAs was ligated to the adaptor provided. Adaptor-ligated tester cDNA was hybridized twice with driver cDNA, followed by selective amplification of differentially expressed cDNAs in a two-step PCR using Advantage cDNA polymerase mix, according to the manufacturer’s instructions. Subtracted cDNAs were sub-cloned into pBluescript II (Stratagene, La Jolla, CA, USA) for sequencing and used as probes in Northern blotting.

### 3.3. RNA Preparation, Northern Blot and RT-PCR

Total RNA extraction from the cells and embryos were performed as described earlier [30]. Poly-A RNA was further extracted from the total RNA using a Dyna-beads mRNA direct kit (Dynal, Oslo, Norway), as per the manufacturer’s instructions. Northern blotting was performed as described earlier [30]. The TSP-1 probe of 0.6 K in size, representing the 3′-UTR, from bp3534 to bp4232, of TSP-1 mRNA (gene bank accession No. M87276), used for Northern blot, was generated via cDNA subtraction. The probes for Runx2 and GAPDH were the same as described earlier [30]. For RT-PCR, cDNA was synthesized from 1 μg of total RNA using AMV Reverse Transcriptase (Promega, Madison, WI, USA) with random primers. Resultant cDNA was used as a template for PCR using Mastermix from Takara Biotech (Dalian, China), as per the manufacturer’s protocol. Primers used are described in Table 1.

### 3.4. Transient Transfection and Luciferase Assay

The 2.8 K mouse TSP-1 promoter in pXP1-luc, described elsewhere [43], was used to study the TSP-1 promoter activity. This vector was co-transfected with either pCMV5 or pCMV5-Runx2 [44] into NIH3T3 cells by the polyfect^R^ transfection reagent (Qiagen, Hilden) for assessing the TSP-1 promoter activity against Runx2. Similarly, the 2.8 K TSP-1 promoter-reporter vector was also co-transfected with pEF-Bos or pEF-Bos/Runx3 for analyzing the Runx3-mediated TSP-1 promoter activity. The luciferase reporter activity was measured 48 h after transfection using a luciferase reporter assay system (Promega) on a TD-20/20 Luminometer (Turner Designs, Sunnyvale, CA, USA).

### 3.5. Chromatin Immunoprecipitation (CHIP)

CHIP was carried out by using a CHIP assay kit (Upstate Biotechnology, Lake Placid, NY, USA), as per the instructions supplied by the manufacturer and the protocol published elsewhere [44]. Runx2 overexpressing C3H10T1/2 stem cells and Runx3 overexpressing B16-F10 melanoma cells were crosslinked, and chromatin was extracted followed by sonication. Immunoprecipitation was carried out by incubating the fragments with anti-flag antibodies (Sigma Aldrich) for flag-Runx2 and flag-Runx3. CHIP assays were also performed in the absence of antibody to control for nonspecific interactions. Amplification was carried out by PCR, and the primers used were: sense: 5′-tccaggcagctggagtcatc-3′; antisense: 5′-aaccatctggctccaggatc-3′.

### 3.6. Western Blot and Immunofluorescence

Cells were lysed in RIPA buffer supplemented with protease inhibitors. Equal amounts of protein were resolved on 10% SDS-PAGE and transferred to PVDF membranes, followed by incubation with mouse monoclonal anti-TSP-1 and anti-β-actin antibodies (Santa Cruz Biotechnology, Santa Cruz, CA, USA). Bound antibodies were visualized by chemiluminescence using the ECL Plus Western Blotting Detection system (GE Healthcare, Piscataway, NJ, USA). Immunofluorescence microscopy was performed as described earlier [44].

## 4. Conclusions

We have identified for the first time that TSP-1 is a putative target gene of the Runt domain transcription factors, Runx2 (critical regulator of bone formation) and Runx3 (tumor suppressor gene), as per both *in vitro* and *in vivo* evidence. Especially, TSP-1 might act as a cancer therapeutic, as it inhibits angiogenesis and slows tumor growth. Since the endogenous levels of TSP-1 are not capable of producing the maximum level of inhibition, therapeutic approaches that are designed to increase TSP-1 levels could be effective for the inhibition of tumor growth and angiogenesis. In this regard, the finding that Runx3 can upregulate TSP-1 expression can be exploited. In all, these findings could be beneficial for the elucidation of the molecular regulatory mechanisms of TSP-1 expression and, finally, for designing therapy against metabolic bone diseases and cancers.

## Figures and Tables

**Table 1:** List of primers used in RT-PCR.

Gene	Forward	Reverse
GAPDH	CTCATGACCACAGTCCATGC	CACATTGGGGGTAGGAACAC
Runx3	CTCGATGGTGGACGTGCTGG	ACCTTGATGGCTCGGTGGTAGG
TSP-1	GAGTGCAAAGAAGTGCCTGATG	GGAATGGACAGTTGTCCCTGTC
